# “Nurses and health professionals facing female genital mutilation: a qualitative study “

**DOI:** 10.1186/s12912-023-01549-6

**Published:** 2023-10-30

**Authors:** MIdoia Ugarte-Gurrutxaga, Victoria- Mazoteras-Pardo, Gonzalo Melgar de Corral, Brígida Molina-Gallego, Laura Mordillo-Mateos, Sagrario Gómez-Cantarino

**Affiliations:** 1 Department of Nursing, Physiotherapy and Occupational Therapy, University of Castilla-La Mancha, Toledo, Spain; 2 Nursing, Pain and Care Research Group, University of Castilla-La Mancha, Toledo, Spain; 3 National Hospital of Paraplegics. Health Service of Castilla-La Mancha, Toledo, Spain

**Keywords:** Female genital mutilation, Nursing, Midwives, Midwifery, Health Professionals

## Abstract

**Background:**

Transnational migratory movements make Spain a country with a very diverse population, including women and girls from countries where Female Genital Mutilation (FGM) is practiced. Given this reality, we set out to carry out a qualitative study to identify the knowledge, attitudes and skills of health professionals regarding FGM.

**Method:**

Qualitative study with a content analysis approach. Forty-seven health professionals with the profiles of Nursing, Family Medicine, Pediatrics, Midwifery and Gynecology and Obstetrics were purposively selected. Data were collected through semi-structured in-depth interviews and focus groups. The qualitative content analysis approach was used for data analysis. The study was conducted in the years 2019 and 2022.

**Results:**

Although most professionals are aware of the current legislation on FGM in Spain, only a few of them are aware of the existence of the FGM prevention protocol in Castilla-La Mancha. This lack of knowledge together with the perception that FGM belongs to the private sphere of women, contributes to the loss of opportunities to identify and prevent FGM.

**Conclusion:**

Health professionals’ training, especially midwives and pediatricians, is essential to the identification and action against Female Genital Mutilation.

## Background

Female genital mutilation (FGM) is defined by the World Health Organization [1] as “all procedures involving partial or total removal of the external female genitalia, or other injury to the female genital organs for non-medical reasons” and is internationally recognised as a violation of girls’ and women’s human rights as it has been enshrined in many international conventions [2]. In fact, in recent years, many destination countries have implemented laws, policies and programs to prevent and respond to FGM [3, 4].

According to the United Nations Children’s Fund [5], there are more than 200 million women and girls worldwide who have been undergone FGM, which has serious consequences for their physical, psychological and social health [6]. There are several reasons behind this practice: hygiene, religion, to secure marriage for girls, to maintain virginity before marriage and to avoid marital infidelity [7].

On the other hand, it is important to bear in mind that the prevalence of FGM is not only marked by geographical barriers, it varies between the ethnic groups present in the 30 countries of sub-Saharan Africa and in some countries of the Middle East and Asia [5]. It should be noted that the demographic weight of the sub-Saharan immigrant population in Spain, with high rates of masculinity, favours family reunification, together with the high fertility rate of African women, and predicts a sharp increase in health centres and schools of girls at risk of FGM.

Although the response to FGM, like all situations of gender-based violence, requires a multi-sectoral approach, the health sector is strategically positioned to prevent FGM [8]. For health professionals, this has meant discovering different cultural realities and facing new care challenges in the context of complex processes of acculturation and social integration [9].

In the literature we reviewed, we found several studies that highlight the shortcomings of health professionals’ lack of knowledge about FGM, which also has a negative impact on the care they provide to women [10–12].

The novelty of this study is that, in addition to knowledge about FGM, the study of the attitudes and behaviours of health professionals towards FGM, particularly those involved in paediatric care and women’s sexual and reproductive health, allows us to identify the key aspects for these professionals to consider that identifying the risk of FGM, preventing it and acting when faced with a case of FGM is a competence that corresponds to them.

Based on this approach, the aim of this study is to explore the attitudes, knowledge and practices of health professionals in relation to FGM.

## Methods

### Study design

This research includes a qualitative methodological perspective using the content analysis method to understand discourses, opinions and underlying ideas. The qualitative analysis steps described by Graneheim and Lundman [13] were followed.

### Participants

Forty-seven health professionals belonging to the Castilla-La Mancha Health Service from the provinces of Toledo, Albacete and Guadalajara, the areas with the highest sub-Saharan African population in Castilla-La Mancha, participated. A purposive sampling method was used to select professional profiles related to pediatrics and sexual and reproductive health: Nursing, Family Medicine, Pediatrics, Midwifery and Gynecology-Obstetrics. Table 1 shows the area of work (primary care and hospital care), sex and profile of the sample.

**Table 1:** Health professionals who have participated in the study

Profile professional	Primary Care		Hospital Care		Total
	W*	M**	W*	M**	%
Nursing	7	4	1		25,5
Family Medicine	7	3	0	0	21,3
Pediatrics	7	4	1	1	27,7
Midwifery	6		2	2	21,3
Gynecology Obstetrics	0	0	2	0	4,2

W*: Woman; M**: Man

In addition to these participants, eight people with the profile designed for the study were asked to take part in the interviews, but they declined on the grounds that they did not have enough time.

### Data collection

Twenty semi-structured in-depth interviews and three focus groups were conducted with 47 health professionals.

All interviews were conducted by telephone after checking availability and obtaining signed informed consent by email from the interviewee. All interviews were conducted by the lead author (MIUG), a nurse and PhD in social and cultural anthropology with research experience and several published qualitative articles. The interviews lasted on average 30–45 min and all started with a general question to establish a close rapport with the participants. The focus groups were conducted face-to-face at the participants’ workplaces and lasted 90 min. Table 2 shows the thematic axes and questions for the interviews and focus groups. It was not considered necessary to pilot test the question scripts for the interviews and focus group discussions.

**Table 2:** Outline of content and question scripts for interviews and focus group

Thematic axes	Questions
Personal data	Sex Education Field of work: Primary Care/Hospital Care Service/Working unit
Knowledge about FGM	Do you know what FGM is? Do you know the different types of FGM? Do you know the countries where it is practised? Do you know the factors that perpetuate it? Do you know the consequences for the health of women who survive this cultural practice? Do you know if there is legislation on FGM in Spain?
Attitudes and behaviours towards the risk of FGM	Have you ever identified a situation of risk of FGM in a girl? What elements have facilitated/hindered you in this identification? If you have identified the risk, how have you acted, what actions have you taken, have you communicated it to another professional, do you know any protocol for action in these cases?
	Have you ever identified a female survivor of FGM in a girl, and what elements have made this identification easier/difficult for you? If you have identified her, how have you acted, what actions have you taken, have you communicated it to another professional, do you know any protocol for action in these cases?
Attitudes and behaviours when faced with a case of FGM	

An exhaustive collection of qualitative data was carried out in terms of transcribing the focus group and interview recordings, as well as recording non-verbal information collected by the observers.

The design of the focus groups ensured the necessary heterogeneity within the homogeneity of belonging to the same population. All professionals worked in primary care. The variables taken into account were age, sex, work experience and professional profile.

Two Primary Care Health Centres were selected, one in Guadalajara (Azuqueca de Henares Health Centre) and one in Toledo (Recas Health Centre), because they cover a geographical area with a higher percentage of sub-Saharan population, which is one of the risk factors for FGM.

Tables 3, 4 and 5 show the composition of each of the three focus groups carried out.

**Table 3:** Composition of Focus Group 1(FG1)- Guadalajara (GU)-PC

Participant	1-Age (years)			2-Sex		3- Work experience (years)		5- Professional profile			
	25–35	36–50	> 50	W*	M **	5–10	> 11	Infirmary	Midwifery	Family Medicine	Pediatrics
FG1-1			**x**	**x**			**X**				**x**
FG1-2		x		x			X				x
FG1-3		x		x		x				x	
FG1-4		x		x			X			x	
FG1-5			x	x			X	x			
FG1-6	x				x	x		x			
FG1-7			x	x			X				x
FG1-8		x			x		X	x			
FG1-9			x		x		X			x	
FG1-10	x			x		x			x		
FG1-11			x	x			X		x		

*Woman; **Man

**Table 4:** Composition of Focus Group 2 (FG2)- Guadalajara (GU)-PC.

Participant	1-Age (years)			2-Sex		3- Work experience (years)		5- Professional profile			
	25–35	36–50	> 50	W*	M **	5–10	> 11	Infirmary	Midwifery	Family Medicine	Pediatrics
FG2-1		x		X			x		x		
FG2-2		x		X			x			x	
FG2-3	x			X		x		X			
FG2-4			x		x		x				x
FG2-5		x		X			x	X			
FG2-6			x		x		x				x
FG2-7		x		X			x			x	
FG2-8				X			x	X			
FG2-9			x	X			x		x		
FG2-10			x		x		x	X			
FG2-11		x		X			x		x		
FG2-12		x		X		x				x	
FG2-13	x			X		x		X			

*Woman; **Man

**Table 5:** Composition of Focus Group 3 (FG3)- Toledo (TO)-PC.

Participant	1-Age (years)			2-Sex		3- Work experience (years)		5- Professional profile			
	25–35	36–50	> 50	W*	M **	5–10	> 11	Infirmary	Midwifery	Family Medicine	Pediatrics
FG3-1		x		X		x			x		
FG3-2			x	X			x			x	
FG3-3			x	X			x	x			
FG3-4		x		X		x					x
FG3-5			x	X		x		x			
FG3-6		x			x	x		x			
FG3-7			x		x		x			x	
FG3-8			x	X			x			x	

*Woman; **Man

We submitted the number of interviews and focus groups to the “data saturation criterion”. Theoretical saturation is reached when the information collected does not contribute anything new to the development of the properties and dimensions of the categories of analysis. The criteria for determining saturation are as follows: (a) the integration and density of the theory (saturation is reached when the greatest number of variations within the theory have been analysed and explained, and (b) when the relationship between the emerging categories obeys a logical explanatory pattern of the investigated problem [14].

### Data analysis

Subsequently, by reading and coding all the information (Atlas-Ti software, Scientific Software Development GmbH, Berlin, Germany), the main dimensions around which the discourse is structured were identified, followed by a distinction between the most relevant aspects of each of the themes, grouping the data collected around categories related to the specific objectives of the study.

We applied a qualitative content analysis approach, which, as a method, is a systematic, objective and flexible resource for understanding a phenomenon that involves labelling and interpreting data in its proper context [15].

The categorisation was carried out in two possible and complementary ways: deductively (those derived from our theoretical framework) and inductively (those that emerged from the discourses of the participants in the study).

### Rigour and trustworthiness

The coding and categorisation were checked by all members of the research team to reach a consensus. We also collaborated with an external reviewer with expertise in conventional content analysis to verify the process of coding, interpreting and categorising data. Data collection was conducted from October to December 2019 in order to have sufficient engagement with the data [16].

In addition, to guarantee rigor, the confidence criteria of Lincoln and Guba [17] were followed. Authors MIU-G and G M-C conducted a peer review, exploring independent perspectives and interpretations throughout the analysis to establish credibility. The transferability of the results was ensured by means of thick description and textual citations. Maintaining an audit trail of study processes established reliability and confirmability [17].

### Ethical consideration

The study was approved from the clinical research ethics committee of the Integrated Healthcare Department of Talavera de la Reina (Toledo, Spain) for this study (CElm Code: 37/2019, of 11 October 2019).

## Results

The following is the procedure for identifying the participants of each speech fragment we have analysed (Table 6).

**Table 6:** Participant identification procedure

The following procedure has been followed for the identification of the issuers of each section of speech incorporated as an example. Each verbatim is followed by a code that consists of four elements: ■ The first is an alphabetical code that identifies whether it is an individual interview or a discussion group. ○ Interview: I ○ Focus Group: FG ■ Guadalajara: GU (1 and 2) ■ Toledo: TO (3)
■ The second identifies the sex of the participant: ○ Women: W ○ Man: M
■ The third part identifies the professional profile of each participant: ○ Nursing: N ○ Midwifery: M ○ Family Medicine: FM ○ Pediatrics: P ○ Gynecology and Obstetrics: GO
■ The third is a number that numerically identifies each participant
■ The fourth is a two-letter code to identify the type of centre in which each participant works: ○ Primary Care: PC ○ Hospital Care: HC

The results are presented below and are structured according to the four themes and categories that emerge from the discourses of the study participants (Table 7).

**Table 7:** Themes and categories identified after the thematic analysis

Topics:	1	2
FGM knowledge, attitudes and behaviours	Knowledge of FGM and current legislation in Spain	Attitudes and behaviour
EMERGING CATEGORIES	Definition and Types of FGM	Faced with the risk of FGM - Ignore risk - Identify risk
	Etiology of FGM	
	Countries where it is practiced	In case of FGM - Ignore - Identify

### Knowledge of FGM

#### Definition and types

Regarding knowledge of FGM, we can conclude that it is known by the study population. Although in many cases it has not been seen or identified directly by professionals, it is known from a theoretical point of view,

*“A surgery, isn’t it? Which is to remove the clitoral area and join the labia together. There are different types, there are… different techniques, that […] sometimes they remove the labia minora, sometimes they also remove the labia minora and the labia majora… There are different degrees, what they do is, above all, well… they do the surgery of the area, come on”.* (IWP13-PC)

*“[…] there are several types of mutilation, but, well, it generally consists of mutilation of the genitals…mostly external (emphasises) of the girl child”. (IWGO9-HC)*

#### Etiology of FGM

As for the reasons for FGM, the predominant idea in the discourses is that this practice is related to culture, religion and even understood as a ritual or traditional rite of certain ethnic groups of origin, they are not very clear:

*“It is the practice that is being carried out due to cultural, religious influence and …. well, it is a little bit the idea that I have transmitted by other patients”.* (FG1-GU)

*”We think they are going on holiday, but in reality, they are going to have this… to have this… ritual practised on them (she emphasises). Of course, this mutilation for them is a rite. […] so to speak, like a rite…normal (emphasising). Something that has to be done.“* (IMM2-PC).

A large majority link the root causes of these practices to violence against women as one of the midwives interviewed said:

*“I consider it gender violence, totally. […] I think it is done… well, to repress her sexually a little bit… So that she is not free in her sexuality, so to speak, and it is a form of violence”.* (IWM1-PC)

#### Countries where it is practiced

From the analysis of the data it is clear that there is knowledge about the different countries or regions where FGM is practised. In the different focus groups and interviews, some regions in Africa and Asia were clearly identified as risk areas, although often it was not possible to specify countries,

*“It is a… practice that is done in sub-Saharan Africa and in some countries in… India and Asia. Well, from the Asian continent”.* (IWP14-PC)

It is very important for professionals to associate the origin of the women as one of the factors that may make them suspect that a woman has been mutilated. This is what one of our interviewees, a midwife in Primary Health Care, said,

*“Well, it is a problem that is usually detected in the sub-Saharan population, black women. In our province we have Senegalese and Malian women who may be susceptible to…. that they have been subjected to this practice”. (IMP8-PC)*

### Attitudes and behaviour

#### In the face of the risk of FGM

##### Ignore risk

According to the discourses, a group of professionals, although aware that there are elements to consider that a girl is at risk of FGM, ignore them. The reasons they give for this are various: lack of skills to deal with it, lack of knowledge about the follow-up of girls at risk and the feeling that it is not within their competence.

One of the reasons that recurrently appears in the discourses is the lack of skills to address the issue and the insecurity that, even if the risk is made visible, the girls will be followed up,

*“If we don’t deal with it, then the same thing can happen to your daughter. So, […] [thinking?] that his daughter has to go through the same thing, that it’s a tradition. So, I think we should act, but… of course, (slight nervous laughter) I don’t know how to do it”. (IMM2-HC)*

We are struck by the comments of two of the paediatricians interviewed, who consider that dealing with FGM does not fall within their competence, although they speak of third parties, with the risk this entails when it comes to identifying girls at risk of being mutilated,

*“The feeling that I have always had is that it does not go with them, it does not go with them because it seems to them that it is something that is already done… that is known to cause many complications in childbirth…. I don’t know if there is any protocol at the gynaecological level to be able to fix… that. But the feeling is… that this doesn’t suit us, nor does it suit us. I don’t know if it’s more a thing of… as it is for adults and everything… as it is the prevention of the child… they see it as something secondary that does not go with them… and that, as if they have problems in childbirth, it is not going to go with them either, that it is a matter for the gynaecologist… I don’t know”. (IWP3-PC)*

*“[…] that it’s not in our, in our practices and we don’t… And we don’t weigh it much, we don’t weigh it much. So, well, sometimes we find it, but we don’t have it internalised within the protocol that we should follow in our search”*. (IMP8-PC)

##### Identifying the risk

Some of the people interviewed commented that it is not easy to identify the risk of female genital mutilation, placing the responsibility on the person who can provide them with the information, often the mother of the girl they see at the clinic,

*“… even the people who are more closed (she emphasises) are perhaps more at risk. The one who is open, and says: I’m certainly not going to do that to my daughter”.* (FG2-GU)

However, they recognise that there are elements that facilitate identification, such as the knowledge that the family is going on a trip to their country of origin, especially if it is a country where FGM is practised,

*“she was a girl […] she was born in Spain, but her parents were from Senegal. […] and I told her all the risks involved: socio-health, psychological…. And then the father […] spoke […] he was a bit… more reticent, because how mothers, […] fathers deal with the issue. Well…I made him understand, I made the…assessment of the girl before he left”.* (IMFM6-PC)

When they attend to a woman whose medical history has already recorded her FGM, it is easier to approach her,

*“I did the other day on a visit for [X] and what they had had was a girl, and the girl was already registered in the system, I did put in notes so that you could see it “Mother with FGM” so that… so that… so that you could be a little more aware”.* (FG3- TO)

Regarding the professional profile and the area where there is a greater possibility of early identification of a girl’s risk of being mutilated, midwives and pediatricians are identified,

*“Those who are most likely to see it are…. (door creaks) [I think?] that midwives”.* (FG2-GU)

*”It’s more the pediatrician that… they find out that they are going on a trip and they may know that they have a girl, but… who knows more, who is closer in that case is the pediatrician”.* (FG3- TO)

Visiting the Preventive Medicine service before the trip (vaccinations) and the Family Medicine Clinic are very good opportunities to get to know the time of the trip to the family’s country of origin,

*“The vast majority come here (Health Centre) because they have to take malaria prophylaxis, […] well, we found out about it. Also to Preventiva go all (emphasising) those who go on a trip to Mali and they don’t leave without the vaccinations, that’s true”.* (high tone of voice) (FG3- TO).

The training received makes it much easier to identify the risk of FGM in girls with mothers who have experienced FGM,

*“I, in my Health Centre, have seen two […] And, then, when they told me the country she was from, that she was Egyptian. I was… I remembered… the percentages, normally, that are given in the countries. Then, my chip was awakened, and I asked her if she was mutilated. As we had already been trained… In Médecins du Monde. They trained us and told us… to ask about it as something normal, like “the cut”.* (Changes voice) *“Were you cut as a child or …. or in your culture you don’t do it? Or some question like that, similar. As a matter of course. And she said yes. Then, this one was going to have a girl. I asked her if…if they wanted to do it to their daughter as well, and she said no. That… her husband’s family didn’t want it* (she emphasises), *and neither did she. And besides, the woman said it like … Very normal”.* (IMM5-PC)

#### Female genital mutilation case

##### Ignore

We see that both in Primary Care and in Specialised Care there are many professionals who either do not detect that a woman has been mutilated or, even though they have identified FGM, they ignore it.

When it comes to the reasons for ignoring, grouped by areas: insecurity (which paralyses), it is not the right time (they are busy with other things), little time to talk due to work overload, it is not considered gender violence, a lot of bureaucracy, lack of empathy, lack of training, the examination of girls in the Child Health Programme is quick (not the same as for boys).

We are concerned that the lack of strategies paralyses someone who has a key role in identifying one of the factors that greatly conditions a girl’s potential risk as the daughter of a woman who has been mutilated. This is not the only testimony we have found in our research, but it is one of the most enlightening, that of a midwife following the pregnancy of a mutilated woman,

*“No, because I feel insecure. I don’t know how to approach it, so…you kind of prefer not to deal with it, to make it as natural as possible (…) It makes me uncomfortable not knowing how to approach it”.* (IMM2-HC)

According to them, at the moment of delivery they are more concerned with “other things” than recording in the medical history whether a woman is mutilated or not, and they even believe that the woman thinks the same way,

*“… the experience was in the process of childbirth. And at that moment, which is a.… a more critical moment, when you are more aware of other things. … maybe about the evolution of the birth, about the baby being well, etcetera, than about dealing with that problem at that moment”.* (IMM2-HC)

*”I take a woman I don’t know at all, she comes to me when she gives birth (…) what she thinks about the least (emphasises) at that moment, what worries her the least is her mutilation”*. (IMM2-HC)

##### Identify

It is curious that in areas where there are large numbers of people from countries where FGM is practised, they say they have not seen any mutilated women,

*“The truth is that I have never come across any… fortunately. (…) And …. and look, I have worked in those years when there was, for example, a lot more immigration, I worked in Vallecas, where there was a lot of… a lot of immigration and a lot… and so on, but I have never come across any case, to be honest”.* (FG1-GU)

According to them, the examination of female genitalia in paediatrics is very fast, so it is very easy for FGM type I to go unnoticed:

*“…genitals is one thing that is explored… quickly, especially in young children because you don’t think that…there’s going to be anything. So… And especially more in women (he emphasises), because in boys, well… well… with phimosis and so on… The testicles, that they are in the pouches… Yes… you are a bit more attentive, but not with girls. So, then, they can go too far”. (IWP14-PC)*

Although they say that there is more tearing when there is FGM, the care provided does not differ from that of other deliveries. Lack of training is again mentioned as an obstacle to the provision of care,

*“So when a woman was torn, and they tend to tear a lot in childbirth… at …. is the… in the area of the clitoris, which is where they have scar tissue and you go to stitch and you see that there is a previous scar…, in whispers I explained to her, I was a bit like saying: “How do we act in a situation that… that you are not prepared to act in?”. (FG3- TO)*

## Discussion

Based on the premise that the number of patients from countries where FGM is practiced in the Health Consulting Services of Castilla-La Mancha is increasing, in terms of quality of care, it is necessary for health professionals to have skills and competencies to give them proper care.

However, although the people we have interviewed seem to have, at least at a theoretical level, knowledge about FGM, its typology and the consequences of this practice on the health of the girls and women who have suffered it. In the study, cultural and religious reasons are mentioned as causes of this practice. However, there is evidence of its existence among groups of different religions [18, 19].

We detected serious shortcomings that greatly condition their ability to detect and adequately treat affected women. The results are in line with several systematic reviews on the perspectives of health professionals, in which a lack of knowledge, competence and understanding of FGM is evidenced [20, 21].

In a study of women’s and men’s perceptions of FGM in Toledo, Castilla-La Mancha, women expressed discomfort with the treatment they received in support meetings [22]. This feeling of mistreatment associated with women’s perception of the low level of clinical and cultural knowledge of professionals in relation to FGM contributes to women feeling disrespected, stigmatized and vulnerable [23, 24].

In the study, the professionals manifested a difficulty when interacting with the women, which may result in a care encounter in which conducting an interview incorrectly in cases of suspected FGM causes discomfort in the patients. Inadequate treatment by professionals may be due to a lack of both linguistic and cultural skills.

This lack leads to misconceptions, lack of awareness, fear and uncertainty about how to talk about FGM and how to offer support to women with FGM. This finding was obtained from among the medical staff, nurses and midwives and although they had questions regarding cultural aspects or sexuality, among others, they did not ask due to concerns about their culturally sensitive appearance.

The systematic review carried out by Evans et al. [25]. also found that for healthcare professionals, feeling confident and able to provide appropriate FGM-related care was strongly related to having the appropriate knowledge, skills and training.

In a recent qualitative study carried out on health professionals of different profiles, they concluded that the existence of a protocol for action and training could be the key tools to take into account to address this problem [26]. It shouldn´t be forgotten that training can improve knowledge of FGM, although not detection, for which action protocols would be necessary [27].

However, as pointed out in the article by Canimas [28] “protocols are necessary, but they can become mechanical algorithms of which professionals are mere executors. A protocol is at the service of the professional, not the other way around” (p.173).

About the professional profile with a greater possibility of early identification of the risk of a girl being mutilated, in the study, specialists related to sexual and reproductive health (midwives, pediatricians and gynaecologists) who carry out their work both in primary care and in the hospital are the main ones identified. Undoubtedly, women’s sexual and reproductive health care and pediatric care are key moments for the detection of cases because they are services of the health system very frequented by migrant women from countries at risk of [29].The need for training perceived by the professionals in our study coincides with that of Kimani et al. [30], who present an experience of training midwives and nurses in which they explain the need and origin of the training of this professional profile for the prevention of FGM. Similar conclusions are reached in the study by Cappon et al. [31] and in that by Sánchez, Caballero, & Moreno [32].

However, the reality is that there is poor or non-existent training at all three competency levels (attitudinal, conceptual and aptitudinal) for professionals in the skills relevant to the treatment of women/girls with FGM-related health complications. As referred in the study by Correa -Ventura & Báez-Quintanaand [33], specific training on FGM increases the detection rate and knowledge about this practice.

On the other hand, the incorporation in the mothers´medical history about FGM, in case that she suffered it, is considered essential to facilitate the risk identification of a girl. However, this record is rare. The difficulties of diagnosis, registration and coding of FGM represent the main obstacle to carry out prevention and care. This situation is constant in several european studies developed among pediatricians, midwives, gynecologists, obstetricians, and physicians who have shown difficulties in detecting, diagnosing, and recording FGM [29, 34, 35].

On the other hand, we have found different attitudes and behaviors on the part of professionals when faced with a case of FGM. We found that both in Primary Care and in Specialised Care there are many professionals who, although they have identified FGM, ignore it, arguing their attitude due to the insecurity created by the lack of knowledge of how to act (lack of training) and the overload of work (care and bureaucratic).

In a survey conducted among midwives in the United States [36], participants showed more accurate knowledge of the types and health consequences of FGM rather than about knowledge of cultural and legal issues.

Another United States study [37] reports that practitioners are unprepared to respond to the many medical, social and mental health consequences for women survivors of FGM.

Uncertainty about the management of the practice is also related to the perception that it is a private matter and is seen as an intrusion into women’s culture. Fear of stigma contributes to avoidance. This attitude may be rooted in the low cultural competence of the professionals in the study. Insecurity about the management of the practice is also linked to the perception, by professionals, that it is a private matter and is seen as an intrusion into women’s culture. They even seem to consider that “it is not their responsibility.“ Fear of stigma contributes to avoidance. This attitude may be rooted in the low cultural competence of study professionals. This situation is also found in the study carried out in the United States by Fay. et al. [38]. It is curious that in areas where there are large numbers of people from countries where FGM is practised, they report not having seen any mutilated women. Similar results are found in the study by Abdulcadir et al. [39]. One of the arguments put forward in our study refers to the lack of attention and the speed with which girls’ genitalia are examined in the Health Programme (unlike boys). For this reason, and in the case of Type I FGM, it goes unnoticed. In our study, professionals commented that, although they identify FGM, they act as if it does not exist. The root of this behavior lies in the insecurity they feel when they do not know how to deal with this issue. They do not even register the mutilation in the mother’s medical history. This not only harms the mother, but also makes it difficult to identify the risk of FGM in their future daughters. In a study conducted in Switzerland, the same results were reached: FGM is not accurately diagnosed, recorded and/or coded [40]. This study concludes that in health centers where specific training is carried out and the use of protocols is implemented, there is a significant increase in the registration of FGM cases in the Clinical History. On the other hand, we found scientific evidence that the existence of a specific marker in the Electronic Medical History to indicate the diagnosis of FGM facilitates the incorporation of this type of gender violence [10].

However, a minority of professionals comment that when they detect a woman who has undergone FGM, the person to whom they inform and refer the case is the Hospital Social Worker, as they consider FGM to be a “social case”. This action serves to prevent FGM among girls who are daughters of mutilated women. In some cases, this preventive action is carried out after having learned about the FGM Prevention Protocol of Castilla-La Mancha [41].

### Limitations

It is pertinent to bear in mind that, although the research is regional in nature, the qualitative phase of the study was carried out in three geographical areas where there is a greater number of people from countries where FGM is practiced. Therefore, we cannot directly extrapolate the results we have obtained from the health professionals in the study to the rest of the professionals who carry out their work in the health services.

Finally, despite the fact that in the development of the interviews we have favored a climate of trust and confidentiality that would allow health professionals to speak and express themselves freely, it is possible that there was a certain effect of social desirability, which may have made their speaches more mediatic. However, to mitigate its possible effects, an attempt has been made to saturate the information in all cases, to obtain solid blocks of content that we could verify in the different speeches.

## Conclusion

This research shows that the approach to FGM in the Health Service can be improved.

The results show that probably due to a lack of knowledge coupled with insecurity about handling an issue (FGM) considered by health professionals as something intimate to women, in many cases it is not reflected in the Medical History of women. Both women that suffered FGM or the risk their daughters have of suffering from it. The fear of stigmatizing women and the lack of intercultural communication skills favor this situation.

Greater training of health professionals in cultural competence would make communication easier and the diagnosis rate of FGM would be higher. We emphasize the importance of midwives and pediatricians in identifying cases of FGM and risk in girls with families from at-risk countries.

This study has been carried out in selected health areas in the Castilla-La Mancha region, so future research should be carried out in other areas. The more information that is collected, the better health policies and programs can be targeted as a fundamental part of efforts to eradicate female genital mutilation.

## Data Availability

The datasets generated and/or analyzed during the current study are not publicly available due some interviews contain information that reveals the identity of individuals but are available from the corresponding author upon reasonable request.
